# A Rare Case of Arterial and Venous Thromboembolism in a Patient With Severe Clostridium difficile Infection

**DOI:** 10.7759/cureus.16103

**Published:** 2021-07-02

**Authors:** Aneesh Kumar, Haider Ghazanfar, Joshua M Davidson

**Affiliations:** 1 Internal Medicine, BronxCare Health System, Bronx, USA

**Keywords:** clostridium difficile, mortality, anticoagulation, thromboembolism, pulmonary embolism, hypercoagulable state

## Abstract

Most cases of Clostridium difficile infection are hospital acquired; but in recent times, the incidence of community-acquired infections has increased. Patients with Clostridium difficile infections are at an increased risk for thrombosis. We report a case of an 82-year-old female who was admitted to the intensive care unit for acute hypoxic respiratory failure and septic shock. She was found to have Clostridium difficile infection at presentation. During the hospitalization, we discovered bilateral lower extremity venous thromboembolism, bilateral pulmonary embolism, multifocal thromboembolic brain infarctions, and acute arterial thromboembolic occlusion of right upper and lower extremities. This patient adds to the list of cases of venous thromboembolism associated with Clostridium difficile infections and is the first case of associated arterial embolic phenomenon. There is a need to further investigate the association of Clostridium difficile infections and thromboembolism.

## Introduction

Clostridium difficile is a gram-positive anaerobic bacterium that colonizes the intestinal tract. Earliest reports of toxin producing Clostridia date back to 1978 [1]. The major risk factors for Clostridium difficile include advanced age, antibiotic use, proton pump inhibitors use, obesity, cirrhosis, inflammatory bowel disease, chemotherapy and immunosuppressive therapy [2]. Severe infections can present as fulminant colitis, characterized by hypotension, shock, ileus and toxic megacolon [3]. We present an unusual case of arterial and venous thromboembolism in a patient with severe Clostridium difficile infection.

## Case presentation

An 82-year-old female presented to our hospital with fever, dry cough, shortness of breath, and poor appetite for three days. She was treated for pneumonia two weeks prior to hospitalization by her primary doctor and had completed a 10-day course of an oral antibiotic. There was no previously reported history of Clostridium difficile infection. She had moved to the United States two years prior. She was ambulatory and functional at baseline.

At the time of presentation, her blood pressure was 89/48 mmHg, pulse rate was 101 beats per minute, respiratory rate was 40 breaths per minute and temperature was 101 ° Fahrenheit. On examination, she was found to be obtunded and not moving any extremities. Her abdomen was soft and distended with reduced bowel sounds. Her peripheral pulses were palpable and her pupils were reactive bilaterally. Her trachea was cannulated for acute respiratory failure. Despite intravenous fluid challenge, she remained hypotensive and was started on norepinephrine for suspected septic shock and admitted to the intensive care unit. She was started on intravenous vancomycin, piperacillin-tazobactam, and azithromycin. Her initial laboratory findings have been mentioned in Table 1.

**Table 1:** Initial laboratory values RT-PCR: reverse transcriptase-polymerase chain reaction; SARS-CoV-2: severe acute respiratory syndrome coronavirus 2.

Parameter	Day 1	Day 2	Reference Range
White blood cell count	23.5	38.9	4.8-10.8 k cells/ul
Neutrophils	92	85	40-70%
Hemoglobin	12.6	11.0	12.0-16.0 g/dL
Serum sodium	156	147	135-145 mEq/L
Serum potassium	6.2	4.1	3.5-5.0 mEq/L
Serum creatinine	2.8	1.1	0.5-1.5 mg/dL
Serum glucose	498	176	70-120 mg/dL
Lactic acid	10.2	1.7	0.5-1.6 mmoles/Liter
Plasma D-dimers	7,629	19,382	0-230 ng/mL
C-reactive protein	255	438	<=5.00 mg/L
Prothrombin time	14.5	12.4	9.9-13.3 second(s)
Partial thromboplastin time	26.1	27.7	27.2-39.6 second(s)
RT-PCR for SARS-CoV-2	Negative	Negative	Negative

**Figure 1:**
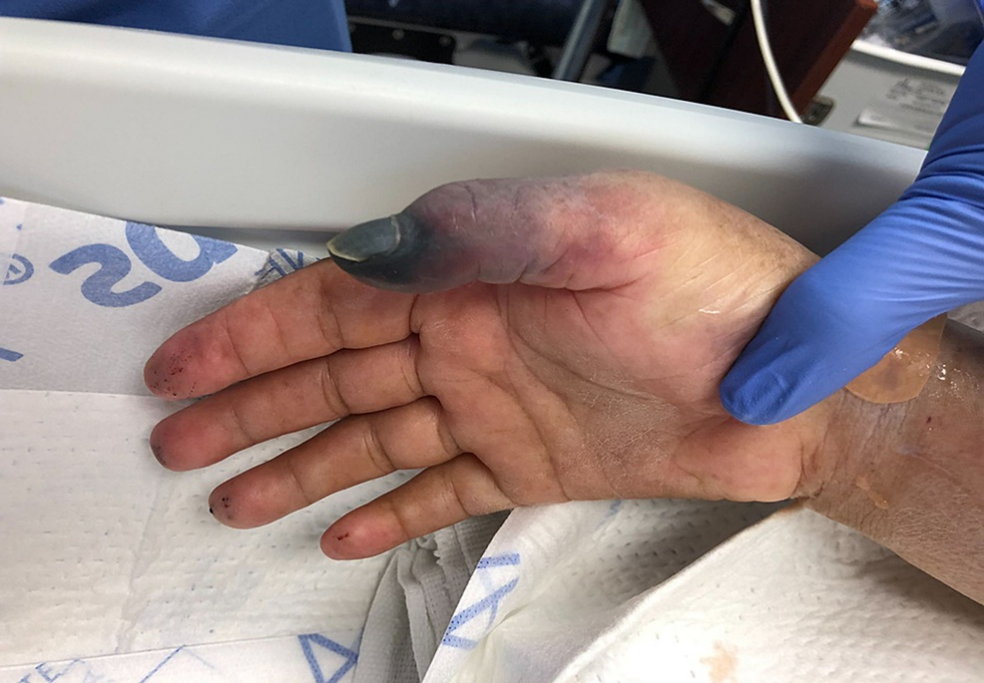
Image of right upper extremity showing gangrenous thumb, second and third digits

**Figure 2:**
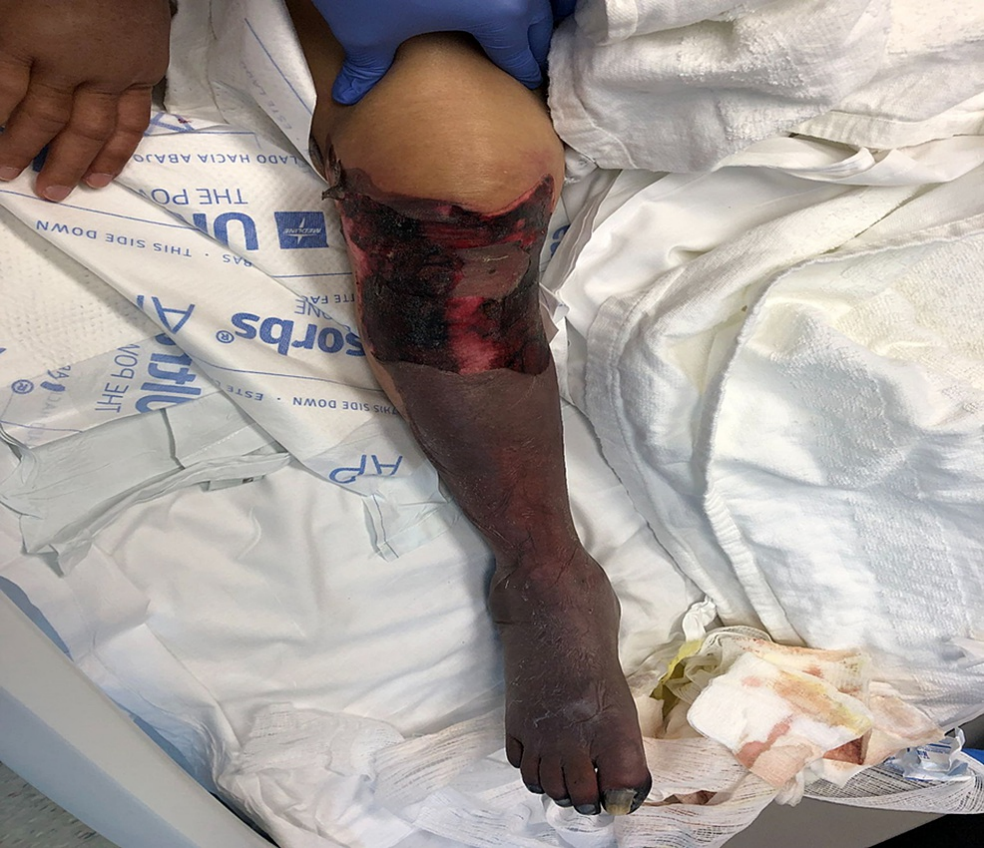
Image of right lower extremity showing gangrenous right leg with a clear demarcation

X-ray imaging of the chest showed diffuse bilateral pulmonary infiltrates. Computed tomography (CT) imaging of her brain showed no acute large vessel distribution infarction, hemorrhage, mass, or mass effect. Venous Doppler ultrasound of her lower extremities showed bilateral deep venous thrombosis and therapeutic subcutaneous enoxaparin was started. She had multiple episodes of watery diarrhea since arrival in the ICU and she was started on oral vancomycin. The stool was sent for analysis and was found to be positive for the glutamate dehydrogenase (GDH) antigen and Clostridium difficile toxin B. She was noticed to have cold mottled skin and decreased peripheral pulses, first in the right upper extremity followed by the right lower extremity. This has been presented in Figures 1-2.

**Figure 3:**
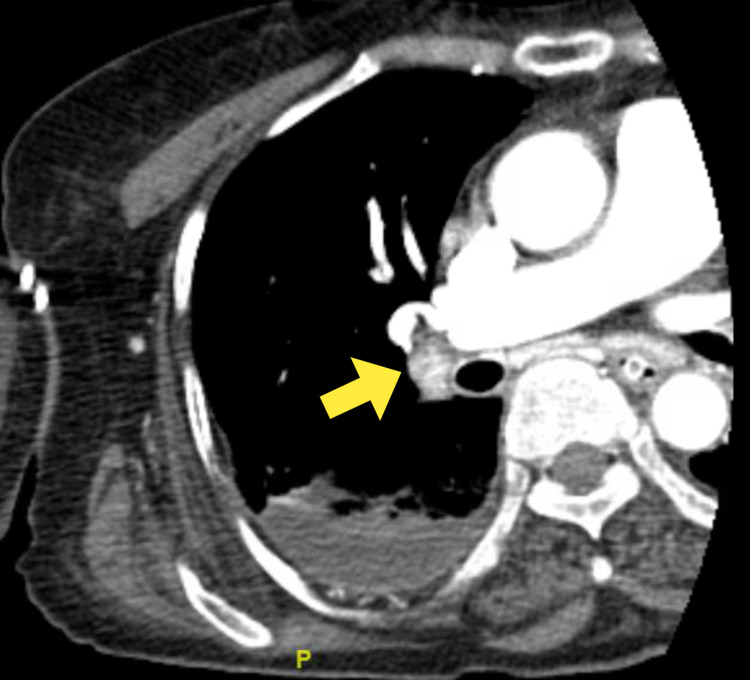
Computed tomography of the chest showing a pulmonary embolism in the right main pulmonary artery (yellow arrow)

CT angiography of the chest revealed a right main pulmonary artery embolism with extension into the lobar branches (Figure 3).

**Figure 4:**
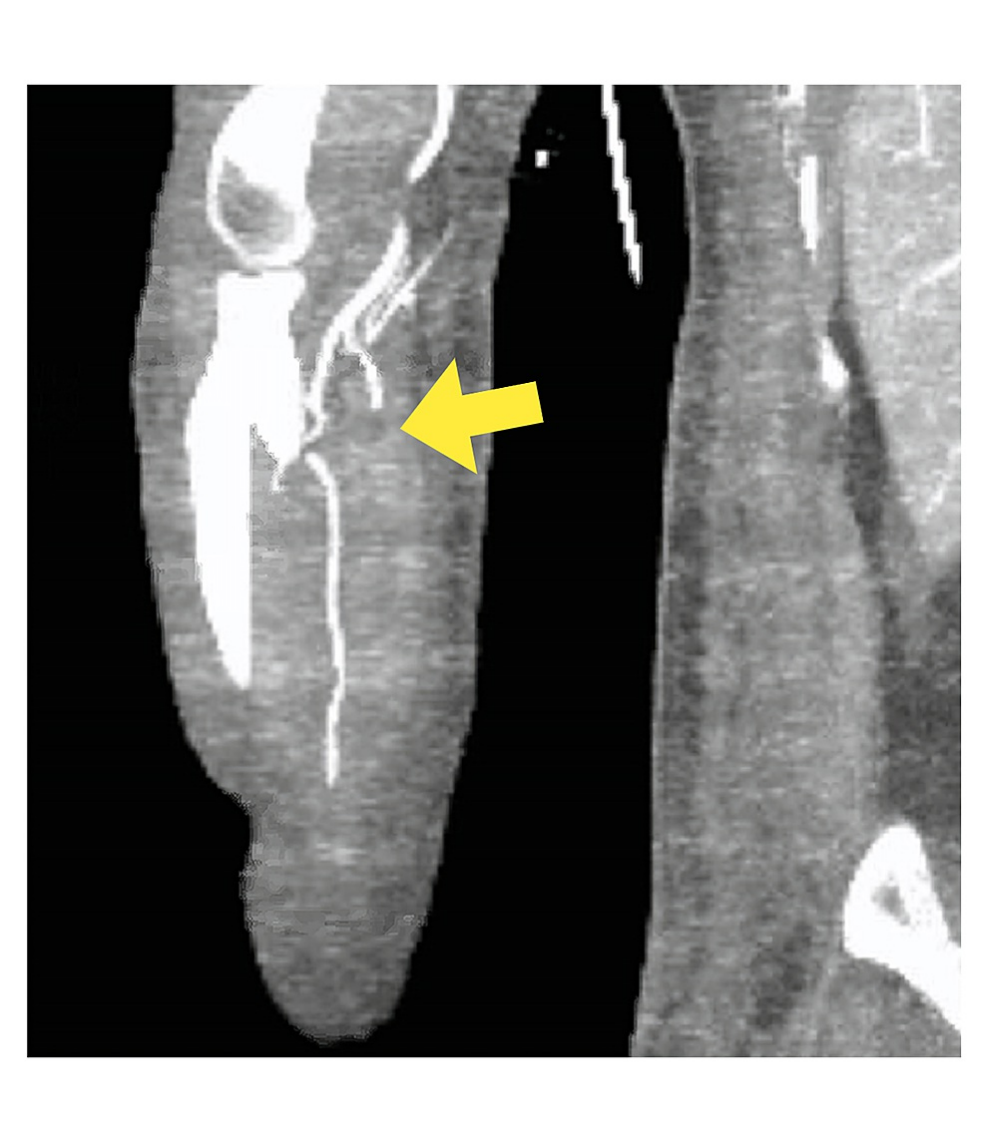
Computed tomography of the right upper extremity showing an occluded right radial artery

CT of her right upper extremity revealed an occluded right radial artery, patent right ulnar artery and the branch vessels in the right wrist and hand appeared to have a distal tapered occlusion, suspicious for distal arterial embolism (Figure 4).

**Figure 5:**
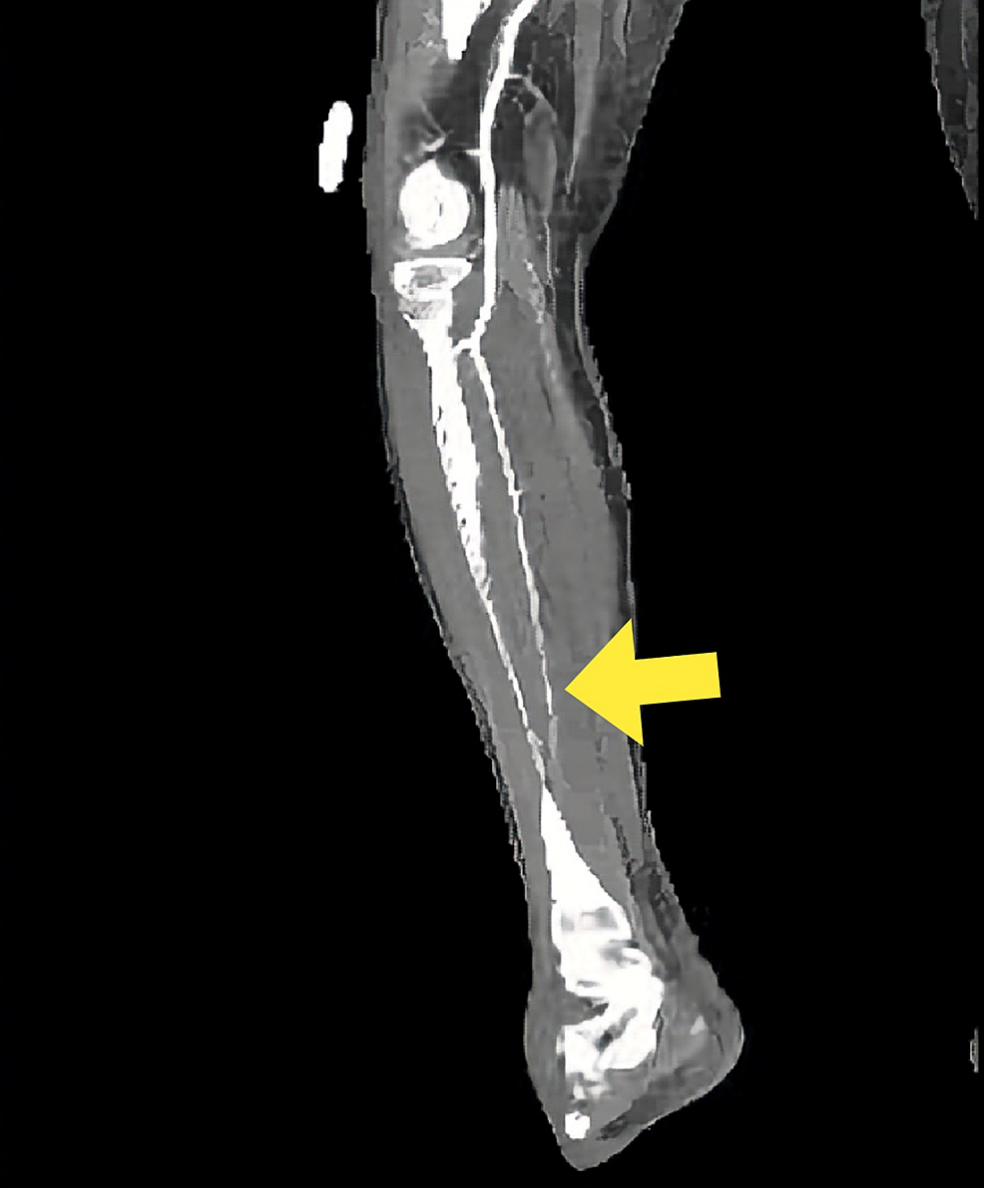
Computed tomography of the right lower extremity showing a tapered distal occlusion of all three blood vessels

CT angiography of her right lower extremity revealed a tapered occlusion of all three trifurcation vessels suspicious for distal thromboembolic occlusion. There was no soft tissue gas or drainable fluid collection in bilateral lower extremities (Figure 5).

The hematology team was consulted, who recommended unfractionated heparin therapy and advised to test for antiphospholipid syndrome. An echocardiogram did not reveal an intracardiac thrombus. Hypercoagulability workup was not remarkable for genetic or other acquired conditions predisposing to thrombosis (Table 2).

**Table 2:** Hypercoagulable workup

Test parameter	Result	Reference
Factor V Leiden mutation analysis	Negative	Negative
Protein C functional assay	87%	70-180%
Protein S functional assay	79%	60-140%
Antithrombin III assay	102%	80-135%
Homocysteine, total	5.0	<10.4umol/l
Heparin-induced thrombocytopenia panel	Negative	Negative
Dilute russell viper venom time confirm	Negative	Negative
Cardiolipin antibody screen	Not detected	Not detected

**Figure 6:**
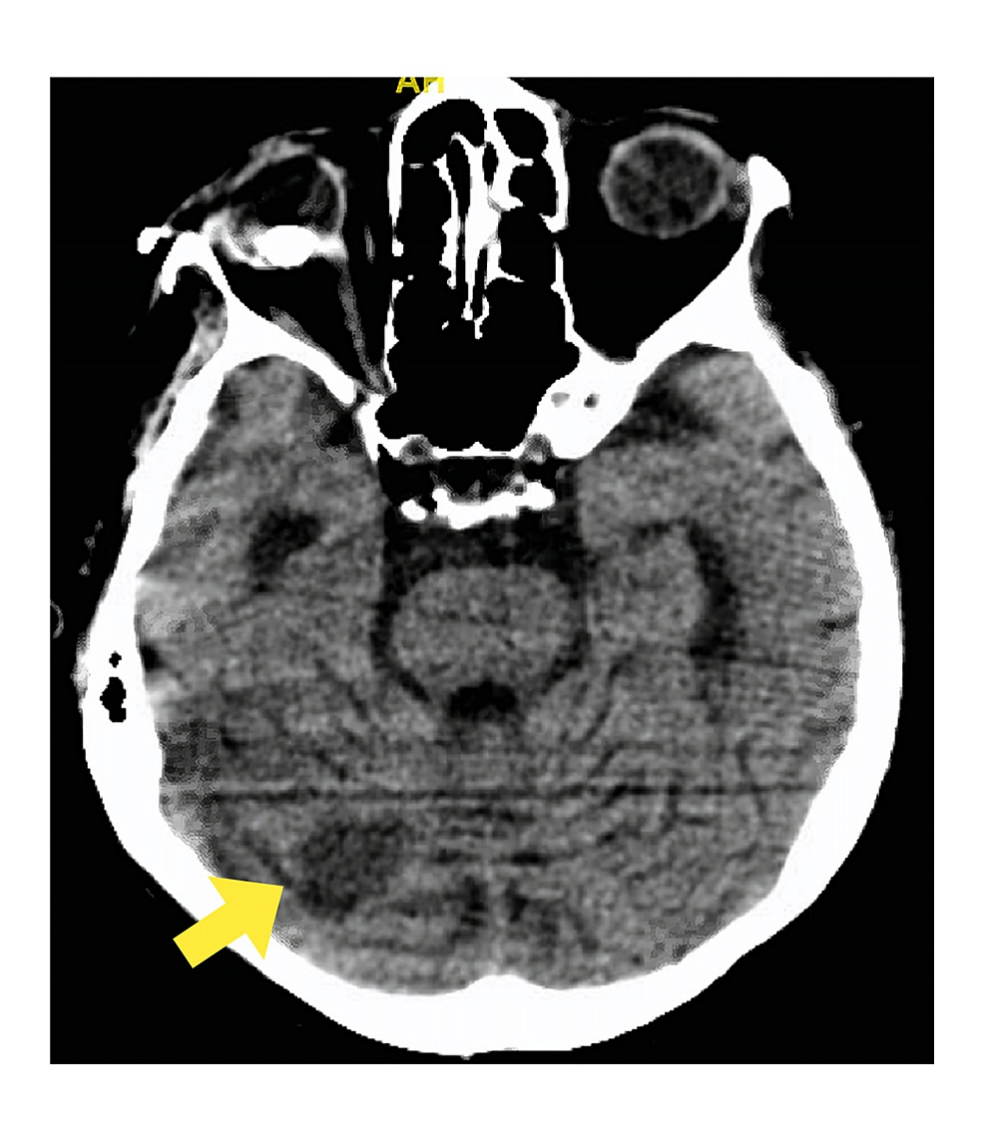
Computed tomography of the brain showing an infarction in the right cerebellum

**Figure 7:**
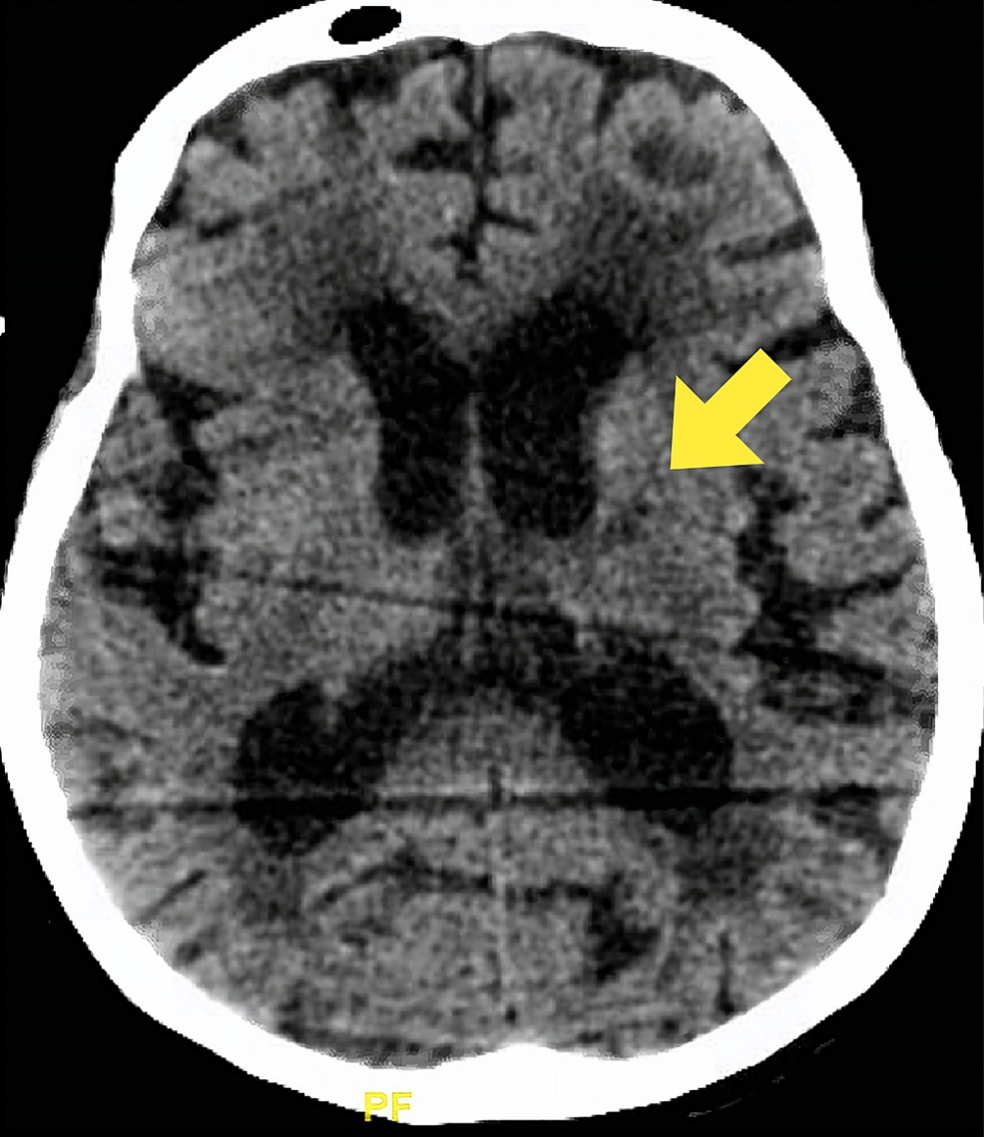
Computed tomography of the brain showing an infarction in the left basal ganglia and thalamus

Multiple attempts to wean her off the ventilator were unsuccessful. On day 5 of hospitalization, repeat CT of the brain showed multifocal infarctions in the right cerebellum and left basal ganglia/thalamus with other smaller foci of watershed infarcts (Figures 6-7).

Her condition continued to deteriorate and her family opted for palliative care.

## Discussion

Clostridium difficile is the leading cause of hospital-acquired diarrhea in the United States of America. According to a study done in 10 states, the estimated incidence of healthcare-associated Clostridium difficile was 73.3 per 100,000 populations and that of community-associated Clostridium difficile infection was 70.4 per 100,000 populations. The national burden of health-care-associated Clostridium difficile infections were found to decrease by 36% from 2011 to 2017, while no change was seen in the prevalence of community-associated Clostridium difficile infection [4]. Clostridium difficile infection is associated with an increased risk of subsequent 30 days, 90 days, and one-year hospitalization. Clostridium difficile infection has also been associated with an increased risk of death (odds ratio (OR): 1.77; 95% confidence interval (CI), 1.74-1.81) [5].

The pathogenesis of Clostridium difficile infection is mediated by the production of two exotoxins; toxin A and toxin B. There have been conflicting reports regarding the individual importance of both toxins in disease. Previous animal studies reported that both toxins act synergistically and that toxin B alone does not cause disease in animals [6]. Gene knockout studies conducted later have shown that toxin B is the key virulence factor [7]. The common symptoms of Clostridium difficile infections are watery diarrhea, low-grade fever, lower abdominal pain, cramping, and anorexia [8]. Severe colitis can present with severe watery diarrhea, diffuse or lower abdominal pain, fever, and abdominal distension [3]. 

**Figure 8:**
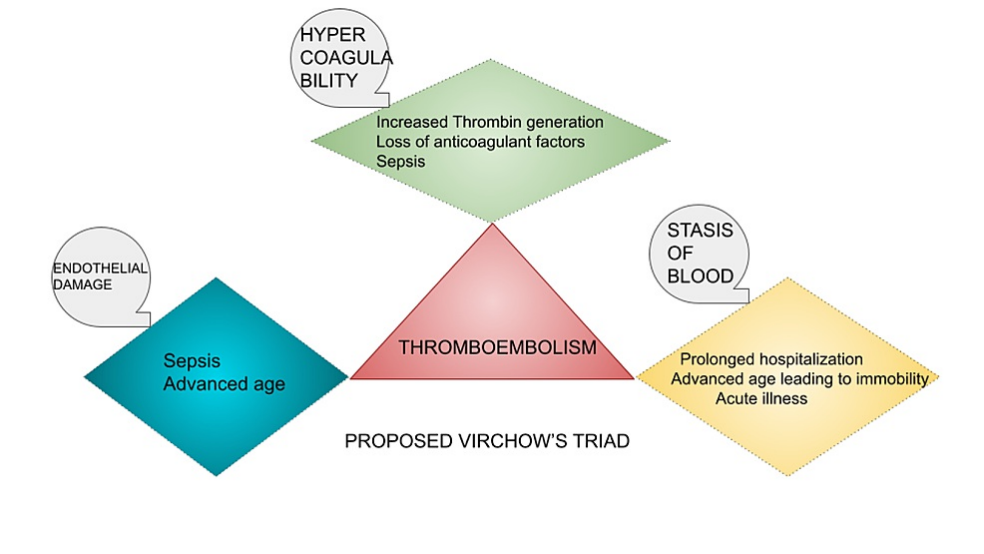
Simple depiction of the proposed Virchow’s triad for thrombotic risk in Clostridium difficile infections Image credits: Aneesh Kumar

There have been reports of increased incidence of venous thromboembolic episodes (VTE) in patients with Clostridium difficile infections [9-11]. According to one study, it was found that patients with a positive Clostridium difficile testing were more likely to have associated VTE (OR of 3.23 (95% CI 1.00, 10.45). It was concluded in the study that the direct causation of the Clostridium difficile infection with VTE seemed less likely, but more so due to prolonged immobilization due to increased lengths of stay [10]. A surgical cohort study that included 1728 surgical patients over a course of 30 months showed an independent association of VTE with Clostridium difficile (OR: 1.87 [1.00 to 3.47], P = .049) [10]. A pilot study described increased thrombin generation seen in patients admitted with Clostridium difficile infections and highlighted this as one of many underlying mechanisms [11]. Based on these reports, a simplified version of Virchow’s triad for VTE in Clostridium difficile is depicted in Figure 8.

The unique aspect of our patient was the development of venous and arterial thromboembolism with no other predisposing risk factor or systemic pathology. She was diagnosed with severe Clostridium difficile infection at admission and did not have a prolonged hospital course to suggest immobilization as a predisposition. Our patient also did not have any history of prior VTE. This is also the first report of widespread arterial thromboembolism with Clostridium difficile infection. There are currently no predictors to predict the risk of thrombosis in Clostridium difficile infection.

## Conclusions

Patients with Clostridium difficile infections are at an increased risk for thrombosis. Given the high morbidity and mortality associated with both of these conditions, it is important to make a prompt diagnosis and to initiate early treatment. There is a need to further investigate the association of Clostridium difficile infection and thrombosis.
